# The impact of tofacitinib on fatigue, sleep, and health-related quality of life in patients with rheumatoid arthritis: a post hoc analysis of data from Phase 3 trials

**DOI:** 10.1186/s13075-022-02724-x

**Published:** 2022-04-05

**Authors:** Susan J. Bartlett, Clifton O. Bingham, Ronald van Vollenhoven, Christopher Murray, David Gruben, David A. Gold, David Cella

**Affiliations:** 1 McGill University, 5252 de Maisonneuve Blvd Ouest, 3D.57, Montreal, QC H4A 3S5 Canada; 2 Johns Hopkins University, Baltimore, MD USA; 3 Amsterdam Rheumatology and Immunology Center, Amsterdam, The Netherlands; 4 Pfizer Inc, Collegeville, PA USA; 5 Pfizer Inc, Groton, CT USA; 6 Pfizer Inc, Montreal, QC Canada; 7 Northwestern University Feinberg School of Medicine, Chicago, IL USA

**Keywords:** Fatigue, Health-related quality of life, Patient-reported outcomes, Rheumatoid arthritis, Sleep, Tofacitinib, Vitality

## Abstract

**Background:**

Fatigue, a common symptom of rheumatoid arthritis (RA), is detrimental to health-related quality of life (HRQoL). We evaluated the impact of tofacitinib on fatigue, sleep, and HRQoL and explored associations between fatigue, related patient-reported outcomes (PROs), and disease activity in RA patients.

**Methods:**

This post hoc analysis pooled data from three Phase 3 studies of tofacitinib (ORAL Scan; ORAL Standard; ORAL Sync) in RA patients. Patients received tofacitinib 5 or 10 mg twice daily, placebo, or adalimumab (active control; ORAL Standard only, not powered for superiority) with conventional synthetic disease-modifying antirheumatic drugs. Assessed through Month (M)12 were changes from baseline in disease activity, Functional Assessment of Chronic Illness Therapy-Fatigue (FACIT-F), Medical Outcomes Study Sleep scale (MOS-SS), and Short Form-36 Health Survey (SF-36) composite/domain scores, and proportions of patients reporting improvements from baseline in FACIT-F total and SF-36 domain scores ≥ minimum clinically important differences (MCIDs) or ≥ population normative values. Pearson correlations examined associations among PROs at M6. Treatment comparisons were exploratory, with *p <* 0.05 considered nominally significant.

**Results:**

Generally, active treatment led to significant improvements from baseline in FACIT-F total, and MOS-SS and SF-36 composite/domain scores vs placebo, observed by M1 and maintained through M6 (last placebo-controlled time point). Through M6, more patients achieved improvements from baseline ≥ MCID and achieved scores ≥ population normative values in FACIT-F total and SF-36 domain scores with tofacitinib vs placebo. Through M12, some nominally significant improvements with tofacitinib vs adalimumab were observed. With active treatment at M6, FACIT-F scores were moderately (0.40–0.59) to highly (≥ 0.60) correlated with SF-36 composite/domain scores (particularly vitality), moderately correlated with most MOS-SS domain scores, and highly correlated with MOS-SS Sleep Problems Index I scores. Disease activity correlations were moderate with FACIT-F scores and low (0.20–0.39) to moderate with SF-36 general health domain/composite scores.

**Conclusion:**

Tofacitinib and adalimumab generally conferred significant, clinically meaningful improvements in fatigue, sleep, and HRQoL (including vitality) vs placebo through M6, with improvements maintained to M12. M6 correlations between FACIT-F, PROs of sleep, HRQoL, and disease activity underscore the interrelatedness of multiple PROs and disease activity in RA.

**Trial registration:**

ClinicalTrials.govNCT00847613 (registered: February 19, 2009); NCT00853385 (registered: March 2, 2009); NCT00856544 (registered: March 5, 2009).

**Supplementary Information:**

The online version contains supplementary material available at 10.1186/s13075-022-02724-x.

## Introduction

Rheumatoid arthritis (RA) is a chronic inflammatory disease associated with significant impairment in health-related quality of life (HRQoL), primarily as a result of pain, impairments in physical function, and fatigue [1–3]. While fatigue affects over 80% of patients with RA, its clinical significance may be overlooked [4, 5]. Fatigue has been described by patients with RA as overwhelming, unpredictable, and severely disabling and is often associated with sleep disturbance, pain, anxiety, and depression [6–8]. The majority of patients take fatigue into account when considering the success of a particular treatment for their RA [9]. Furthermore, in a recent study examining patient-reported outcome (PRO) preferences in patients with rheumatic diseases, participants ranked fatigue as the most important measure for tracking, with this preference particularly significant in patients with RA [10]. Yet, the complexity of the fatigue experience in patients with RA, encompassing physical, cognitive, daily living, and emotional impacts, can make it difficult to manage [6].

Many factors contribute to fatigue in RA, including those related to the disease (disease activity, inflammation, medication side effects), patients’ pain, mood, and ability to function, and other individual patient factors, including comorbidities and lifestyle [11]. Also, sleep disturbance is common in patients with RA, with over half of patients reporting suboptimal sleep duration [8, 12], further contributing to increased fatigue [11]. The Canadian Early Arthritis Cohort (CATCH), an observational cohort of patients with early inflammatory RA, examined fatigue within the first year following diagnosis and noted that, while there was a significant decrease in fatigue at the time of first remission, maximal improvements in fatigue lagged behind this first remission by 6 months [13]. Risk factors for fatigue included more active disease, greater pain, obesity, depression, and poor sleep [14, 15]. Early use of methotrexate was associated with improved fatigue [14], with optimal weight management also thought to play an important role in attenuating persistent fatigue [14, 15].

Randomized controlled trials of tofacitinib in patients with active RA have shown sustained improvements in the PROs of pain, sleep, fatigue, and HRQoL [16–22]. Improvements in fatigue were also observed alongside improvements in the Short Form-36 Health Survey (SF-36) bodily pain and vitality domains [17]. However, the associations between fatigue and related PROs in tofacitinib-treated patients with RA have not previously been investigated.

This analysis of data pooled from the Phase 3 ORAL Scan, ORAL Standard, and ORAL Sync clinical trials further evaluates the impact of tofacitinib, administered in combination with background conventional synthetic (cs) disease-modifying antirheumatic drugs (DMARDs), on fatigue, sleep, and HRQoL. Additionally, we explore associations between fatigue and related PROs, and between fatigue, HRQoL, and disease activity in patients with RA with previous inadequate response to at least one csDMARD or biologic (b)DMARD.

## Methods

### Design and participants

This was an exploratory post hoc analysis of data pooled from three global Phase 3 randomized controlled trials (ORAL Scan [NCT00847613]; ORAL Standard [NCT00853385]; ORAL Sync [NCT00856544]) of tofacitinib administered in combination with csDMARDs in patients with active RA [23–25]. The trials were of 12 (ORAL Standard and ORAL Sync) or 24 months’ (ORAL Scan) duration, and the individual study designs have been described in detail previously [23–25].

Eligible patients were ≥ 18 years of age with a diagnosis of active RA, as defined according to the American College of Rheumatology 1987 Revised Criteria [26]. Active disease was classified as ≥ 6 (ORAL Standard and ORAL Scan) or ≥ 4 (ORAL Sync) tender/painful joints, ≥ 6 or ≥ 4 swollen joints (68- and 66-joint counts, respectively), and an erythrocyte sedimentation rate ≥ 28 mm/h (Westergren method) or a C-reactive protein level > 7 mg/L. Key exclusion criteria included current treatment with antirheumatic agents other than csDMARDs, including bDMARDs, and current infection or evidence of active or inadequately treated infection with *Mycobacterium tuberculosis*. All patients were required to have had a prior inadequate response to methotrexate (ORAL Scan and ORAL Standard) or at least one csDMARD or bDMARD (ORAL Sync).

All studies were conducted in compliance with the Declaration of Helsinki, the International Council for Harmonisation Good Clinical Practice Guidelines in the European Community, and local country regulations. The final protocol, any amendments, and informed consent documentation were reviewed and approved by the Institutional Review Boards and the Independent Ethics Committees of the investigational centers. All patients provided written informed consent.

### Treatment

Patients received tofacitinib 5 mg twice daily (BID; approved dose) or 10 mg BID, adalimumab 40 mg once every 2 weeks (Q2W; ORAL Standard only as an active control), or placebo. Patients in the placebo group who did not have a 20% reduction in the number of swollen and tender joints after 3 months (considered non-responders) were blindly advanced to receive either tofacitinib 5 or 10 mg BID. After 6 months, all patients assigned to placebo were blindly advanced to receive either tofacitinib 5 or 10 mg BID. All patients continued to receive stable background doses of csDMARD therapy (specifically methotrexate in ORAL Scan and ORAL Standard). In patients receiving concomitant methotrexate, stable doses of methotrexate were administered at 15–25 mg/week; stable doses < 15 mg/week were allowed but only if there were safety issues at higher doses.

### Outcomes

In this analysis, change from baseline at months 1, 3, 6, and 12 was reported for two disease activity outcomes (Disease Activity Score in 28 joints, erythrocyte sedimentation rate [DAS28-4(ESR)] and Clinical Disease Activity Index [CDAI]), and the following PROs: Functional Assessment of Chronic Illness Therapy-Fatigue (FACIT-F) total score (range 0–52, higher scores indicate less fatigue) [27, 28]; Medical Outcomes Study Sleep scale (MOS-SS) six domain scores (range 0–100 [except quantity of sleep, where the maximum score was 24 (hours)], higher scores indicate greater sleep disturbance), and MOS-SS Sleep Problems Index I (six items) and II (nine items) summary composite scores [29, 30]; and SF-36 eight domain scores, and Physical Component Summary (PCS) and Mental Component Summary (MCS) composite scores (range 0–100, higher scores indicate better HRQoL; for this analysis, the domain/composite scores were rescaled to have a mean of 50 and standard deviation of 10, normed to the US-based population).

Changes from baseline were compared with published values for minimum clinically important differences (MCIDs) for DAS28-4(ESR) (≥ 1.2-point decrease from baseline) [31], FACIT-F total score (≥ 4.0-point increase from baseline) [27], and SF-36 domain and composite scores (≥ 5.0-point and ≥ 2.5-point increase from baseline, respectively) [32]. No MCID values have been determined for the MOS-SS. While MCID values have been determined for the CDAI [33], thresholds vary according to baseline disease activity and are not reported here. The proportions of patients reporting improvements from baseline in DAS28-4(ESR) score, FACIT-F total score, and SF-36 domain scores ≥ MCID were analyzed at months 1, 3, 6, and 12.

Also, the proportions of patients reporting FACIT-F total scores ≥ the population normative value (defined as an average score of 43.5) [34] and SF-36 domain scores ≥ the population normative value (based on average age- and gender-matched norms of the SF-36 Scoring Manual [35]), were analyzed at months 1, 3, 6, and 12 for those patients with FACIT-F total scores greater than, or SF-36 domain scores less than, normative at baseline.

Finally, correlations between FACIT-F total score and other PROs (MOS-SS and SF-36 composite and domain scores) and disease activity (DAS28-4[ESR] and CDAI), and between HRQoL measures (SF-36 general health domain and composite scores) and disease activity at month 6 were evaluated.

### Statistical analyses

The full analysis set included all patients who underwent randomization and received at least one dose of study medication. For patients who were originally receiving placebo before switching to tofacitinib (either at month 3 or 6), their post-switch observations were excluded from the analysis.

Continuous endpoints were summarized by treatment and visit using descriptive statistics and were analyzed using a repeated-measures mixed-effects linear model, with study, visit, treatment group, geographic region, and baseline value of the dependent variable as main effects, and a two-way interaction effect of visit-by-treatment. All visits available from each study were included up to month 12. This longitudinal model implicitly imputed for missing data under the assumption of missing at random. Estimates of mean change from baseline for each treatment group were derived from the model as least squares (LS) means and corresponding standard errors. Binary endpoints (rates of MCID and normative values achieved) were also summarized by treatment and visit using descriptive statistics and were analyzed at each visit using a logistic regression model, with study, treatment group, and geographic region as effects. Missing values were not imputed.

Given that this was a post hoc analysis and, as such, was not powered to test non-inferiority or superiority of one treatment over another, any between-group comparisons were considered exploratory, with *p* < 0.05 considered nominally significant.

Pearson correlations were conducted (pairwise with FACIT-F) within each treatment group at month 6, with no imputation of missing values. Descriptive *p* values (indicating if the correlation was not zero) are reported. No adjustments to *p* values for multiple comparisons were made.

## Results

### Patients

A total of 2265 patients were included in the analysis (tofacitinib 5 mg BID, *n* = 826; tofacitinib 10 mg BID, *n* = 821; adalimumab 40 mg Q2W, *n* = 199; placebo, *n* = 419). Patient demographics and baseline disease characteristics were generally similar across treatment groups (Table 1). Most patients were white and female and had long-standing RA (mean range across treatment groups: 8.2–9.1 years since diagnosis). Mean age range across treatment groups was 52.2–53.2 years. At baseline, disease activity was high (mean CDAI range 35.7–37.1) across treatment groups. The proportions of patients receiving concomitant methotrexate at baseline were 100%, 96.3%, and 79.0% for ORAL Scan, ORAL Standard, and ORAL Sync, respectively.

**Table 1:** Demographic and baseline disease characteristics across treatment groups^a^

	Tofacitinib 5 mg BID (*N =* 826)	Tofacitinib 10 mg BID (*N =* 821)	Adalimumab 40 mg Q2W (*N =* 199)	Placebo (*N =* 419)
Age, mean (SD) years	53.2 (11.7)	52.2 (11.6)	52.7 (11.6)	52.7 (11.9)
Female, *n* (%)	694 (84.0)	684 (83.3)	157 (78.9)	335 (80.0)
Race, *n* (%)				
Asian	267 (32.3)	265 (32.3)	27 (13.6)	137 (32.7)
Black	22 (2.7)	18 (2.2)	3 (1.5)	9 (2.1)
White	475 (57.5)	459 (55.9)	148 (74.4)	239 (57.0)
Other	62 (7.5)	79 (9.6)	21 (10.6)	34 (8.1)
Geographic region, *n* (%)				
Canada/Europe	279 (33.8)	272 (33.1)	114 (57.3)	144 (34.4)
Latin America	112 (13.6)	107 (13.0)	21 (10.6)	53 (12.6)
USA	138 (16.7)	133 (16.2)	30 (15.1)	76 (18.1)
Other^b^	297 (36.0)	309 (37.6)	34 (17.1)	146 (34.8)
Disease duration, mean (SD) years	8.3 (7.5)	8.7 (8.0)	8.2 (7.6)	9.1 (8.6)
DAS28-4(ESR), mean (SD)	6.4 (1.0)^e^	6.4 (1.0)^f^	6.4 (0.9)^g^	6.3 (1.0)^h^
CDAI, mean (SD)	36.3 (12.1)^i^	36.1 (12.2)^e^	37.1 (12.8)	35.7 (12.6)^j^
HAQ-DI, mean (SD)	1.4 (0.7)^k^	1.4 (0.7)	1.5 (0.6)	1.4 (0.7)^l^
FACIT-F total score, mean (SD)	28.6 (10.8)^m^	29.0 (10.3)^e^	27.9 (10.1)	30.4 (10.1)^l^
MOS-SS domain, mean (SD)				
Sleep adequacy	46.1 (28.4)^n^	45.1 (27.4)^o^	44.1 (27.9)^p^	47.0 (25.9)^l^
Awaken short of breath/with headache	18.5 (25.0)^n^	17.1 (23.6)^o^	20.0 (24.4)^p^	16.7 (22.5)^l^
Sleep disturbance	43.3 (26.8)^n^	42.0 (25.8)^e^	46.6 (26.3)^p^	40.1 (26.6)^l^
Sleep quantity (hours)	6.6 (1.7)^k^	6.6 (1.5)^q^	6.7 (1.8)^r^	6.7 (1.5)^s^
Snoring	34.0 (31.6)^q^	31.5 (31.4)^e^	34.7 (30.7)^r^	31.4 (29.3)^t^
Somnolence	35.7 (22.1)^i^	35.5 (21.9)^o^	33.4 (19.8)^r^	34.9 (19.9)^l^
MOS-SS Sleep Problems Index I score^c^, mean (SD)	39.8 (20.9)^i^	39.5 (19.4)^o^	41.0 (19.7)^p^	37.6 (18.6)^l^
MOS-SS Sleep Problems Index II score^d^, mean (SD)	41.2 (20.3)^i^	40.8 (19.1)^e^	43.2 (19.5)^p^	39.2 (18.7)^l^
SF-36 domain score, mean (SD)				
Physical functioning	32.3 (9.7)^u^	31.9 (9.7)	31.7 (8.9)	32.9 (10.0)^j^
Role-physical	34.1 (9.5)^u^	34.1 (9.5)	34.8 (8.7)	35.1 (9.7)^j^
Bodily pain	33.6 (7.4)^u^	34.1 (7.6)	33.1 (7.3)	34.8 (7.5)^j^
General health	34.7 (9.1)^u^	35.3 (8.8)	35.2 (8.0)	35.9 (8.3)^j^
Vitality	40.7 (9.9)^u^	41.5 (9.3)	39.9 (9.6)	42.3 (9.5)^j^
Social functioning	36.6 (11.0)^u^	37.7 (11.1)	36.2 (11.6)	38.4 (11.5)^j^
Role-emotional	35.2 (13.0)^u^	35.2 (13.1)	35.6 (12.1)	36.6 (13.1)^j^
Mental health	39.7 (11.8)^u^	40.4 (11.0)	39.7 (11.3)	41.6 (11.1)^j^
SF-36 MCS score, mean (SD)	40.7 (12.0)^u^	41.5 (11.3)	40.6 (11.7)	42.6 (11.5)^j^
SF-36 PCS score, mean (SD)	32.8 (7.9)^u^	32.8 (7.7)	32.7 (6.8)	33.5 (7.5)^j^
PtGA VAS, mean (SD)	58.9 (22.8)^m^	57.9 (23.1)^v^	57.1 (22.3)	55.6 (22.7)^j^
Pain VAS, mean (SD)	58.1 (22.9)^u^	58.3 (22.9)	56.3 (22.0)	55.8 (22.9)^j^
CRP levels, mean (SD) mg/L	16.1 (19.9)^u^	17.3 (23.4)	17.4 (22.5)	15.3 (16.6)

Data were pooled from Phase 3 ORAL Scan, ORAL Standard, and ORAL Sync study datasets and are presented for the full analysis set

^a^All treatments were administered in combination with background conventional synthetic disease-modifying antirheumatic drugs

^b^Countries in “Other” were Australia, China, India, Japan, Malaysia, Philippines, Republic of Korea, Taiwan, and Thailand

^c^Based on six items of the MOS-SS: How often over the past 4 weeks did you … have trouble falling asleep; awaken during sleep; awaken short of breath/with headache; get enough sleep to feel rested upon waking; get amount of sleep needed; have trouble staying awake?

^d^Based on the six items stated in footnote u and three additional items of the MOS-SS: How often over the past 4 weeks did you … feel that your sleep was not quiet; feel drowsy during day; how long did it usually take to fall asleep?

^e^ *N =* 818; ^f^ *N =* 813; ^g^ *N =* 192; ^h^ *N =* 413; ^i^ *N =* 822; ^j^ *N =* 418; ^k^ *N =* 824; ^l^ *N =* 417; ^m^ *N =* 825; ^n^ *N =* 823; ^o^ *N =* 819; ^p^ *N =* 198; ^q^ *N =* 817; ^r^ *N =* 197; ^s^ *N =* 415; ^t^ *N =* 416; ^u^ *N =* 825; ^v^ *N =* 820

*BID*, twice daily; *CDAI*, Clinical Disease Activity Index; *CRP*, C-reactive protein; *DAS28-4(ESR)*, Disease Activity Score in 28 joints, erythrocyte sedimentation rate; *FACIT-F*, Functional Assessment of Chronic Illness Therapy-Fatigue; *HAQ-DI*, Health Assessment Questionnaire-Disability Index; *MCS*, Mental Component Summary; *MOS-SS*, Medical Outcomes Study Sleep scale; *PCS*, Physical Component Summary; *PtGA*, Patient Global Assessment of Arthritis; *Q2W*, once every 2 weeks; *SF-36*, Short Form-36 Health Survey; *SD*, standard deviation; *VAS*, visual analog scale

### Changes in disease activity up to month 12

**Fig. 1:**
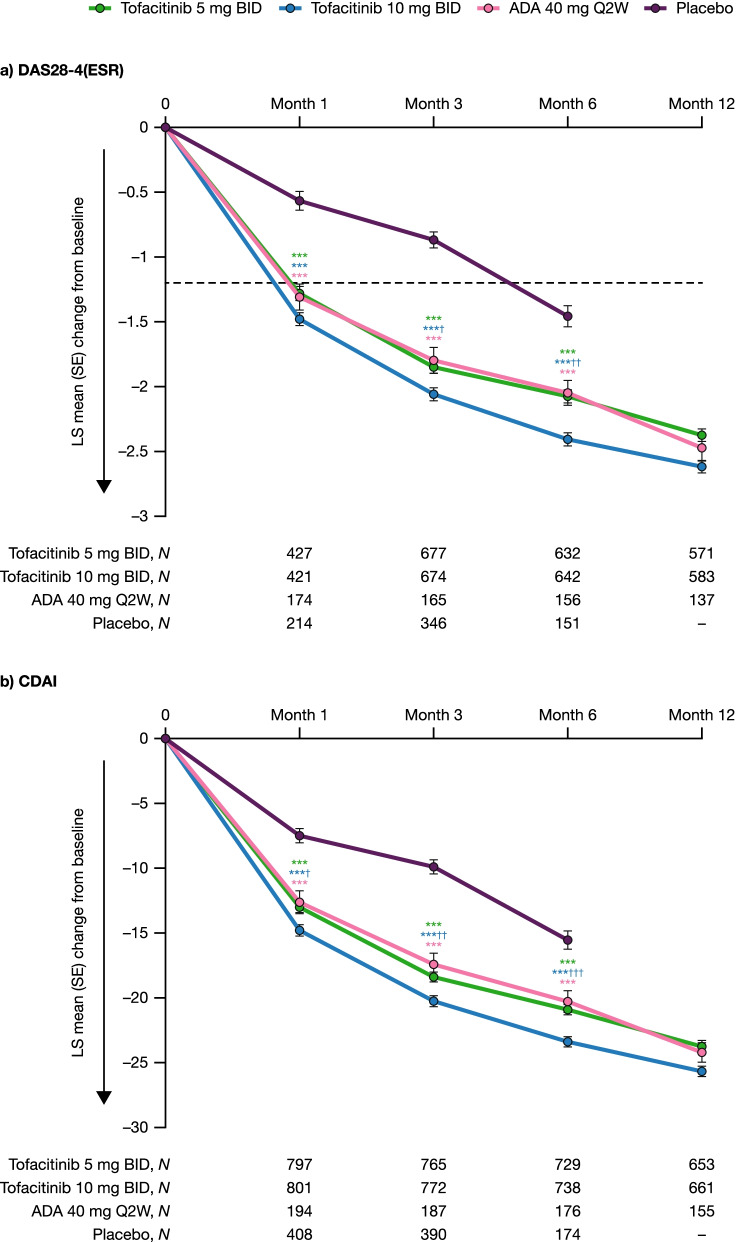
Changes from baseline in disease activity up to month 12. LS mean change from baseline in **a** DAS28-4(ESR) and **b** CDAI to month 12 across treatment groups^a^ pooled from Phase 3 ORAL Scan, ORAL Standard, and ORAL Sync study datasets (full analysis set). ^a^All treatments were administered in combination with background conventional synthetic disease-modifying antirheumatic drugs. ^***^ *p* < 0.001 for tofacitinib and adalimumab vs placebo; ^†^ *p* < 0.05 and ^††^ *p* < 0.01 for tofacitinib vs adalimumab. The horizontal dashed lines represent the MCIDs. The arrows on the *y*-axes indicate the direction of improvement. ADA, adalimumab; BID, twice daily; CDAI, Clinical Disease Activity Index; DAS28-4(ESR), Disease Activity Score in 28 joints, erythrocyte sedimentation rate; LS, least squares; MCID, minimum clinically important difference; Q2W, once every 2 weeks; SE, standard error

Across active treatment groups (tofacitinib [both doses] and adalimumab), significant improvements from baseline in DAS28-4(ESR) and CDAI were observed vs placebo as early as month 1 and were maintained through month 6 (all *p* < 0.001; Fig. 1). Improvements in DAS28-4(ESR) were ≥ MCID with tofacitinib and adalimumab by month 1 (Fig. 1a). There were significantly greater improvements with tofacitinib 10 mg BID vs adalimumab in DAS28-4(ESR) at months 3 and 6 (Fig. 1a; all *p* < 0.05) and in CDAI at months 1, 3, and 6 (Fig. 1b; all *p* < 0.05).

### Changes in PROs up to month 12

**Fig. 2:**
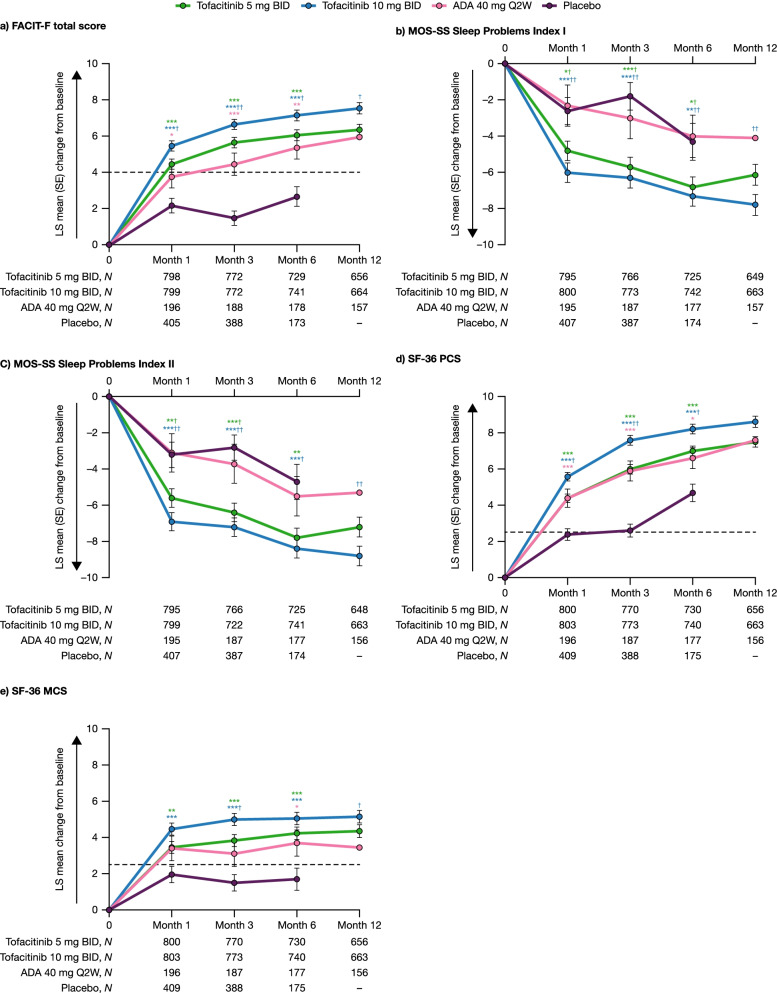
Changes from baseline in PROs up to month 12. LS mean change from baseline in **a** FACIT-F total score, **b** MOS-SS Sleep Problems Index I score^a^, **c** MOS-SS Sleep Problems Index II score^b^, **d** SF-36 PCS score, and **e** SF-36 MCS score to month 12 across treatment groups^c^ pooled from Phase 3 ORAL Scan, ORAL Standard, and ORAL Sync study datasets (full analysis set). ^a^Based on six items of the MOS-SS: How often over the past 4 weeks did you … have trouble falling asleep; awaken during sleep; awaken short of breath/with headache; get enough sleep to feel rested upon waking; get amount of sleep needed; have trouble staying awake? ^b^Based on the six items stated in footnote a and three additional items of the MOS-SS: How often over the past 4 weeks did you … feel that your sleep was not quiet; feel drowsy during day; how long did it usually take to fall asleep? ^c^All treatments were administered in combination with background conventional synthetic disease-modifying antirheumatic drugs. ^*^ *p* < 0.05, ^**^ *p* < 0.01, and ^***^ *p* < 0.001 for tofacitinib and adalimumab vs placebo; ^†^ *p* < 0.05 and ^††^ *p* < 0.01 for tofacitinib vs adalimumab. The horizontal dashed lines represent the MCIDs. The arrows on the *y*-axes indicate the direction of improvement. ADA, adalimumab; BID, twice daily; FACIT-F, Functional Assessment of Chronic Illness Therapy-Fatigue; LS, least squares; MCID, minimum clinically important difference; MCS, Mental Component Summary; MOS-SS, Medical Outcomes Study Sleep scale; PCS, Physical Component Summary; PRO, patient-reported outcome; Q2W, once every 2 weeks; SE, standard error; SF-36, Short Form-36 Health Survey

Significant improvements from baseline in FACIT-F total score (Fig. 2a) were observed with both tofacitinib doses (all *p* < 0.001) and adalimumab (all *p* < 0.05) vs placebo at months 1, 3, and 6. Improvements in FACIT-F total score were ≥ MCID with both tofacitinib doses by month 1 and adalimumab by month 3. Further, improvements in FACIT-F total score were significantly greater with tofacitinib 10 mg BID vs adalimumab at months 1, 3, 6, and 12 (all *p* < 0.05).

Significant improvements from baseline in MOS-SS Sleep Problems Index I and II (Fig. 2b, c) were observed with both tofacitinib doses, but not adalimumab, vs placebo at months 1, 3, and 6 (all *p* < 0.05). Improvements in MOS-SS Sleep Problems Index I and II were also significantly greater with both tofacitinib doses vs adalimumab at months 1 and 3 (all *p* < 0.05) and with tofacitinib 10 mg BID vs adalimumab at months 6 and 12 (*p* < 0.01). In addition, improvements in MOS-SS Sleep Problems Index I were significantly greater with tofacitinib 5 mg vs adalimumab at month 6 (*p* < 0.05).

Significant improvements from baseline in SF-36 PCS score (Fig. 2d) were observed with both tofacitinib doses and adalimumab vs placebo at months 1, 3, and 6 (all *p* < 0.05). Significant improvements in SF-36 MCS score (Fig. 2e) were observed with both tofacitinib doses vs placebo at months 1, 3, and 6 (all *p* < 0.01), while significance was observed with adalimumab vs placebo at month 6 only (*p* < 0.05). Improvements in SF-36 PCS and MCS scores were ≥ MCID with both tofacitinib doses and adalimumab by month 1. Additionally, improvements in SF-36 PCS and MCS scores were significantly greater with tofacitinib 10 mg BID vs adalimumab at months 1, 3, and 6 (all *p* < 0.05), and at months 3 and 12 (*p* < 0.05), respectively.

### Changes in PRO domains up to month 12

Overall, significant improvements from baseline were observed with tofacitinib vs placebo for some MOS-SS domains at some time points (Supplementary Fig. 1a–f). Through month 6, adalimumab did not demonstrate significance in improvements vs placebo for any MOS-SS domain (Supplementary Fig. 1a–f).

At month 1, significant improvements from baseline in MOS-SS sleep adequacy, sleep disturbance, sleep quantity, and somnolence domains were observed with tofacitinib 10 mg BID vs placebo (all *p* < 0.05; Supplementary Fig. 1a, c, d, and f), and in the sleep disturbance domain with tofacitinib 5 mg BID vs placebo (*p* < 0.05; Supplementary Fig. 1c). Also, significant improvements in MOS-SS sleep adequacy and somnolence domains were observed with both doses of tofacitinib vs adalimumab (*p* < 0.05; Supplementary Fig. 1a and f).

At month 3, significant improvements across all MOS-SS domains were observed with both doses of tofacitinib vs placebo (all *p* < 0.05), except snoring (tofacitinib 5 and 10 mg BID) and awaken short of breath/with headache (tofacitinib 5 mg BID) (Supplementary Fig. 1a–f and Table 2; refer to Supplementary Table 1 for simple mean changes in these parameters). Three MOS-SS domain scores (sleep adequacy [tofacitinib 5 mg BID], sleep disturbance, and somnolence [tofacitinib 10 mg BID]) showed significantly greater improvements vs adalimumab [all *p* < 0.05; Supplementary Fig. 1a, c, and f, and Table 2]).

**Table 2:** LS mean change from baseline in PRO domain scores at month 3 across treatment groups^a^

Outcome	Tofacitinib 5 mg BID (*N =* 826)			Tofacitinib 10 mg BID (*N =* 821)			Adalimumab 40 mg Q2W (*N =* 199)			Placebo (*N =* 419)		
	*n*	LS mean change	SE	*n*	LS mean change	SE	*n*	LS mean change	SE	*n*	LS mean change	SE
**MOS-SS domain scores**												
Sleep adequacy	768	7.56^***†^	0.86	773	6.67^**^	0.86	187	3.37	1.81	387	1.86	1.19
Awaken short of breath/with headache	768	0.20	0.69	773	−1.76^*^	0.69	187	−1.07	1.47	387	0.55	0.96
Sleep disturbance	767	−7.58^*^	0.68	772	−8.72^**†^	0.68	187	−4.66	1.43	387	−5.25	0.94
Sleep quantity (hours)	771	0.18^*^	0.04	771	0.27^**^	0.04	186	0.16	0.09	386	0.01	0.06
Snoring	761	−0.65	0.78	771	0.09	0.77	186	0.76	1.64	385	−0.57	1.07
Somnolence	767	−5.25^***^	0.62	773	−5.91^***†^	0.62	186	−3.03	1.32	387	−0.26	0.86
**SF-36 domain scores**												
Physical functioning	772	4.77^***^	0.30	774	6.67^***††^	0.30	188	4.44^**^	0.64	389	2.16	0.42
Role-physical	773	5.42^***^	0.31	775	7.28^***††^	0.31	188	5.37^**^	0.66	389	2.50	0.43
Bodily pain	772	7.41^***^	0.30	775	9.43^***††^	0.30	188	7.19^***^	0.62	389	3.54	0.41
General health	772	4.73^***^	0.26	774	5.59^***^	0.26	187	4.55^***^	0.65	389	1.65	0.36
Vitality	773	5.77^***^	0.31	775	6.65^***†^	0.31	188	5.02^**^	0.65	389	2.18	0.42
Social functioning	773	5.11^***†^	0.33	775	6.31^***††^	0.33	188	3.51	0.71	389	2.26	0.46
Role-emotional	772	3.79^**^	0.39	774	6.24^***†^	0.39	188	4.09^*^	0.83	388	1.93	0.54
Mental health	773	3.93^***^	0.33	775	5.03^***^	0.33	188	3.54^*^	0.69	389	1.59	0.45

Data were pooled from Phase 3 ORAL Scan, ORAL Standard, and ORAL Sync study datasets, and are presented for the full analysis set

^a^All treatments were administered in combination with background conventional synthetic disease-modifying antirheumatic drugs

^*^ *p* < 0.05, ^**^ *p* < 0.01, and ^***^ *p* < 0.001 for tofacitinib and adalimumab vs placebo; ^†^ *p* < 0.05 and ^††^ *p* < 0.01 for tofacitinib vs adalimumab

*BID*, twice daily; *LS*, least squares; *MOS-SS*, Medical Outcomes Study Sleep scale; *PRO*, patient-reported outcome; *Q2W*, once every 2 weeks; *SE*, standard error; *SF-36*, Short Form-36 Health Survey

At month 6, the MOS-SS sleep disturbance domain showed significant improvement with tofacitinib 10 mg BID vs placebo (*p* < 0.05; Supplementary Fig. 1c), and the MOS-SS somnolence domain showed significant improvements with both tofacitinib doses vs adalimumab (all *p* < 0.05; Supplementary Fig. 1f).

At month 12, the MOS-SS sleep adequacy, sleep disturbance, sleep quantity, and somnolence domains showed significantly greater improvements with tofacitinib 10 mg BID vs adalimumab (all *p* < 0.05; Supplementary Fig. 1a, c, d, and f).

Across active treatment groups, improvements from baseline in all SF-36 domains generally exceeded MCID by month 12, with MCID achieved by month 6 in most domains (Supplementary Fig. 2a–h). At month 1, significant improvements across all SF-36 domains were observed with both tofacitinib doses vs placebo (all *p* < 0.05), except SF-36 role-emotional (tofacitinib 5 mg BID), and several SF-36 domains showed significant improvements with adalimumab vs placebo (all *p* < 0.05; Supplementary Fig. 2a–h). Also, SF-36 bodily pain and vitality domains were significantly improved with tofacitinib 10 mg BID vs adalimumab (*p* < 0.05; Supplementary Fig. 2c and e).

At month 3, all SF-36 domains significantly improved with both tofacitinib doses and adalimumab vs placebo (all *p* < 0.05), except for SF-36 social functioning, which was not significantly improved with adalimumab vs placebo (Supplementary Fig. 2a–h and Table 2; refer to Supplementary Table 1 for simple mean changes in these parameters). Most SF-36 domains showed significantly greater improvement with tofacitinib 10 mg BID vs adalimumab at this time point.

Similarly, at month 6, all SF-36 domains significantly improved with both tofacitinib doses and adalimumab vs placebo (all *p* < 0.05; Supplementary Fig. 2a–h), except for SF-36 physical functioning, social functioning, and role-emotional, which were not significantly improved with adalimumab vs placebo. Also, at month 6, several SF-36 domains showed significantly greater improvements with tofacitinib 10 mg BID vs adalimumab.

Finally, at month 12, improvements in SF-36 social functioning and role-emotional domains were significantly greater with tofacitinib 10 mg BID vs adalimumab (all *p* < 0.05; Supplementary Fig. 2f and g).

### Proportions of patients reporting improvements from baseline ≥ MCID in PROs up to month 12

**Fig. 3:**
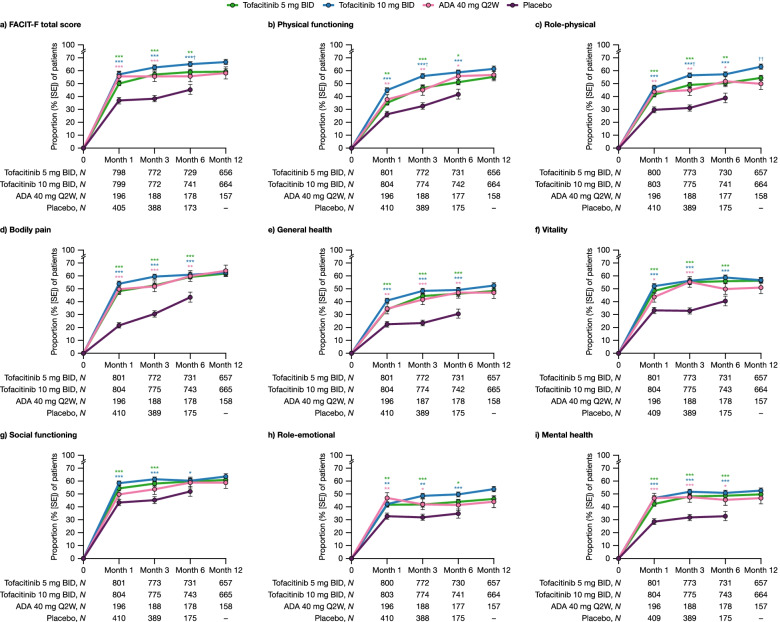
Proportions of patients reporting improvements from baseline ≥ MCID in PROs to month 12. Proportions of patients reporting LS mean change from baseline ≥ MCID in **a** FACIT-F total score and **b–i** SF-36 domain scores, to month 12 across treatment groups^a^ pooled from Phase 3 ORAL Scan, ORAL Standard, and ORAL Sync study datasets (full analysis set). ^a^All treatments were administered in combination with background conventional synthetic disease-modifying antirheumatic drugs. ^*^ *p* < 0.05, ^**^ *p* < 0.01, and ^***^ *p* < 0.001 for tofacitinib and adalimumab vs placebo; ^†^ *p* < 0.05 and ^††^ *p* < 0.01 for tofacitinib vs adalimumab. ADA, adalimumab; BID, twice daily; FACIT-F, Functional Assessment of Chronic Illness Therapy-Fatigue; MCID, minimum clinically important difference; PRO, patient-reported outcome; Q2W, once every 2 weeks; SE, standard error; SF-36, Short Form-36 Health Survey

Through month 6, significantly higher proportions of patients receiving either tofacitinib dose reported improvements from baseline in FACIT-F total scores ≥ MCID vs placebo (all *p* < 0.01; Fig. 3a). This trend was also seen with adalimumab at months 1 and 3 (all *p* < 0.0001; Fig. 3a). At month 6, the proportion of patients reporting improvements ≥ MCID was significantly higher with tofacitinib 10 mg BID compared with adalimumab (*p* < 0.05; Fig. 3a).

Similarly, through month 6, significantly higher proportions of patients receiving either tofacitinib dose reported improvements from baseline ≥ MCID in all SF-36 domains vs placebo, except the SF-36 social functioning domain at month 6, where significance was only reached in patients receiving tofacitinib 10 mg BID (all *p* ***<*** 0.05; Fig. 3b–i). Generally, similar trends were seen through month 6 with adalimumab. At month 3, the proportions of patients reporting improvements from baseline ≥ MCID in the SF-36 physical functioning and role-physical domains were significantly higher with tofacitinib 10 mg BID vs adalimumab, with significance also seen between these treatment groups at month 12 (for the SF-36 role-physical domain only; Fig. 3b, c).

### Proportions of patients achieving normative values in PROs up to month 12

**Fig. 4:**
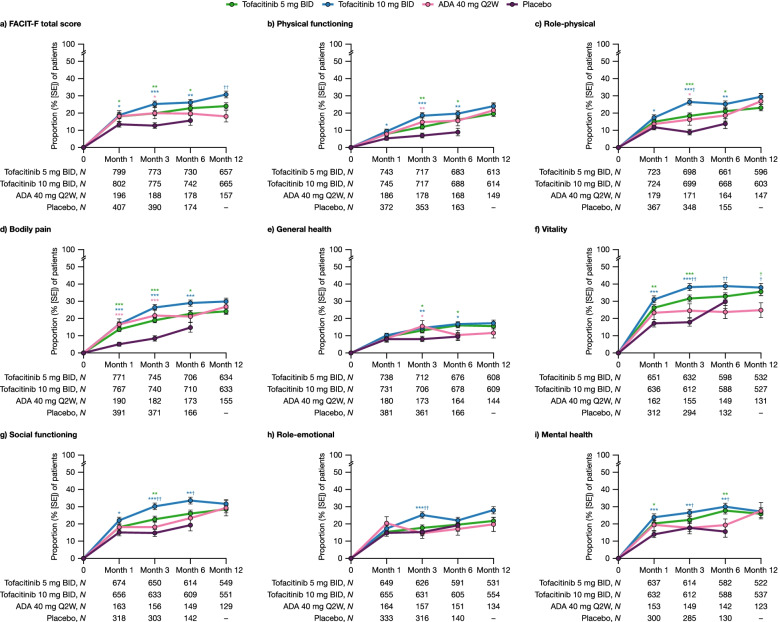
Proportions of patients achieving normative values in PROs up to month 12. Proportions of patients who met or exceeded population normative values for **a** FACIT-F total score and **b–i** SF-36 domain scores, to month 12 across treatment groups^a^ pooled from Phase 3 ORAL Scan, ORAL Standard, and ORAL Sync study datasets (full analysis set). ^a^All treatments were administered in combination with background conventional synthetic disease-modifying antirheumatic drugs. Patients were not normative at baseline. ^*^ *p* < 0.05, ^**^ *p* < 0.01, and ^***^ *p* < 0.001 for tofacitinib and adalimumab vs placebo; ^†^ *p* < 0.05 and ^††^ *p* < 0.01 for tofacitinib vs adalimumab. ADA, adalimumab; BID, twice daily; FACIT-F, Functional Assessment of Chronic Illness Therapy-Fatigue; PRO, patient-reported outcome; Q2W, once every 2 weeks; SE, standard error; SF-36, Short Form-36 Health Survey

At months 1, 3, and 6, significantly more patients receiving either tofacitinib dose met or exceeded normative values for FACIT-F total scores vs placebo (Fig. 4a). The proportion of patients receiving adalimumab who met or exceeded normative values for FACIT-F total scores was significantly higher vs placebo at month 3 only. The proportion of patients who met or exceeded normative values in FACIT-F total scores at month 12 was significantly greater with tofacitinib 10 mg BID compared with adalimumab (Fig. 4a).

At month 1, differences vs placebo in the proportions of patients receiving active treatment who met or exceeded normative values for SF-36 domain scores reached significance for the domains of physical functioning, role-physical, and social functioning (tofacitinib 10 mg BID only; Fig. 4b, c, and g), vitality and mental health (both tofacitinib doses; Fig. 4f, i), and bodily pain (all active treatments; Fig. 4d). At month 3, significantly more patients receiving either tofacitinib dose met or exceeded normative values for SF-36 domains of physical functioning, role-physical, bodily pain, general health, vitality, and social functioning, vs placebo (all *p* < 0.05; Fig. 4b–g). Also, at month 3, significantly more patients receiving tofacitinib 10 mg BID met or exceeded normative values for SF-36 subdomains of role-emotional and mental health vs placebo (*p* < 0.01; Fig. 4h, i). At month 6, significantly more patients receiving either tofacitinib dose met or exceeded normative values for SF-36 domains of physical functioning, role-physical, bodily pain, general health, and mental health vs placebo (all *p* < 0.05; Fig. 4b–e and i). The proportions of patients who met or exceeded normative values for SF-36 vitality, social functioning, and mental health domains were significantly higher with tofacitinib 10 mg BID vs adalimumab at months 3 and 6, as were the proportions of patients who met or exceeded normative values for SF-36 role-physical and role-emotional domains at month 3 (all *p* < 0.05; Fig. 4c and f–i). At month 12, the proportions of patients who met or exceeded normative values for SF-36 vitality were significantly higher with both tofacitinib doses vs adalimumab (all *p* < 0.05; Fig. 4f).

### Correlations between FACIT-F and other PROs and disease activity, and SF-36 general health domain/composite scores and disease activity at month 6

At month 6 and across active treatment groups, SF-36 domain and composite scores were moderately (0.40–0.59) to highly (≥ 0.60) correlated with FACIT-F total scores (Table 3). Correlations between FACIT-F total scores and MOS-SS domain scores were moderate to very low (< 0.20), depending on the individual domain, while MOS-SS Sleep Problems Index I scores were highly correlated with FACIT-F total scores across active treatments (Table 3). Generally, similar trends were observed with placebo (Table 3).

**Table 3:**
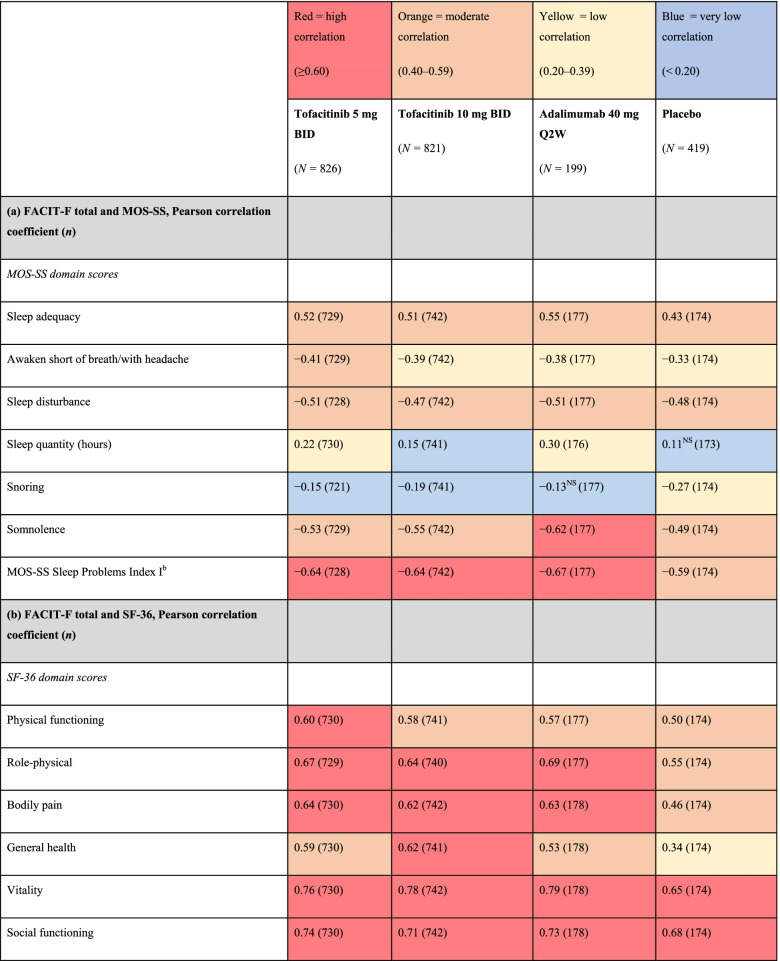

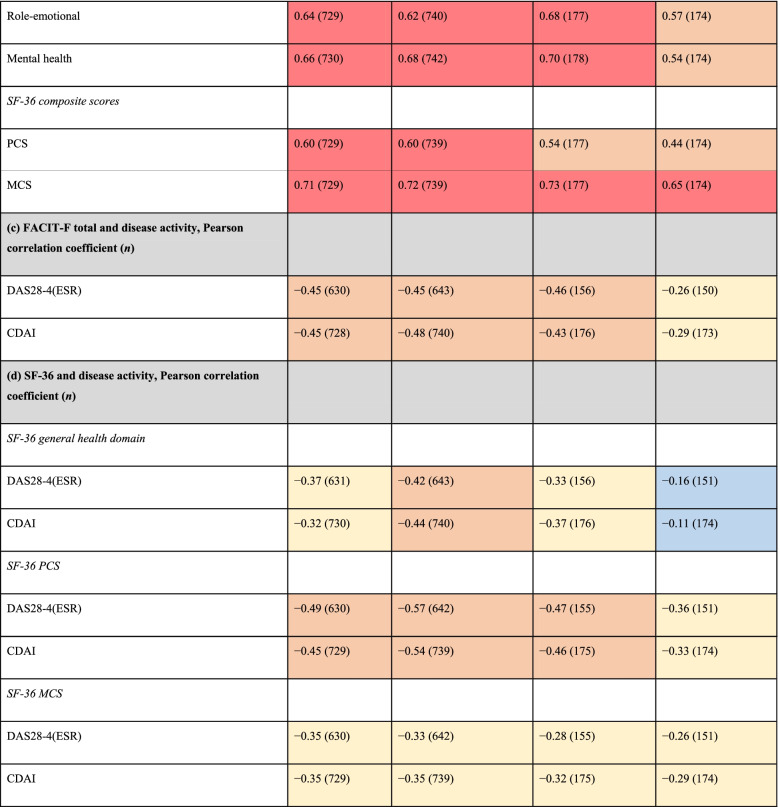
Associations between FACIT-F and (a) MOS-SS, (b) SF-36, and (c) disease activity, and between (d) SF-36 and disease activity at month 6 across treatment groups^a^

Data were pooled from Phase 3 ORAL Scan, ORAL Standard, and ORAL Sync study datasets, and are presented for the full analysis set

^a^All treatments were administered in combination with background conventional synthetic disease-modifying antirheumatic drugs

^b^Based on six items of the MOS-SS: How often over the past 4 weeks did you … have trouble falling asleep; awaken during sleep; awaken short of breath/headache; get enough sleep to feel rested upon waking; get amount of sleep needed; have trouble staying awake?

All correlations differed from zero at *p* < 0.001, except where noted as not significant

*BID*, twice daily; *CDAI*, Clinical Disease Activity Index; *DAS28-4(ESR)*, Disease Activity Score in 28 joints, erythrocyte sedimentation rate; *FACIT-F*, Functional Assessment of Chronic Illness Therapy-Fatigue; *MCS*, Mental Component Summary; *MOS-SS*, Medical Outcomes Study Sleep scale; *NS*, not significant; *PCS*, Physical Component Summary; *Q2W*, once every 2 weeks; *SF-36*, Short Form-36 Health Survey

At month 6, DAS28-4(ESR) and CDAI scores were moderately correlated with FACIT-F total scores across active treatment groups, while in the placebo group, low (0.20–0.39) correlations were observed (Table 3). Low to moderate correlations between SF-36 general health scores, as well as SF-36 PCS and MCS scores, and DAS28-4(ESR) and CDAI scores were reported in the active treatment groups; very low to low correlations were reported in the placebo group (Table 3).

## Discussion

In this exploratory post hoc analysis, we examined pooled efficacy data from the Phase 3 ORAL Scan, ORAL Standard, and ORAL Sync randomized controlled trials of more than 2000 patients with RA and a history of inadequate response to methotrexate (ORAL Scan and ORAL Standard) or at least one csDMARD or bDMARD therapy (ORAL Sync) to assess the impact of tofacitinib administered in combination with csDMARDs on PROs relating to fatigue, sleep, and HRQoL. Additionally, we investigated the correlations between fatigue and related PROs and disease activity in these patients. While both tofacitinib 5 and 10 mg BID data are reported here, it should be noted that tofacitinib 5 mg BID is the approved dose for RA.

Clinically meaningful improvements (≥ MCID) in disease activity were observed as early as month 1 and maintained through month 12 in tofacitinib-treated patients, with significance vs placebo demonstrated through month 6 (i.e., the end of the placebo-controlled period). Treatment with tofacitinib led to sustained improvements in fatigue, sleep, and HRQoL (significance vs placebo generally demonstrated through month 6). Notably, clinically meaningful improvements (≥ MCID) in composite measures of fatigue and HRQoL (per FACIT-F and SF-36 PCS/MCS, respectively) were observed by month 1. Furthermore, more patients treated with tofacitinib achieved improvements from baseline ≥ MCID and ≥ normative values in fatigue and SF-36 domains compared with placebo.

While the original studies were not designed or powered to show non-inferiority or superiority of tofacitinib compared with the active control (tumor necrosis factor inhibitor [TNFi] adalimumab), and statistical associations do not prove causality, exploratory comparisons between treatment groups were made with *p <* 0.05 considered nominally significant. Overall, similar observations were made for both tofacitinib doses (5 and 10 mg BID) and generally for adalimumab, with key differences as follows.

Compared with adalimumab, tofacitinib 10 mg BID was associated with significantly greater improvements in disease activity and fatigue through month 12. Notably, tofacitinib was associated with greater improvements in sleep vs adalimumab, evidenced by the significantly greater improvements in MOS-SS Sleep Scale Problems Index I and II scores generally observed with both tofacitinib doses vs adalimumab through months 1–6. Numerical differences favoring tofacitinib 5 mg BID vs adalimumab were observed with both sleep scores at month 12, while significance was maintained with tofacitinib 10 mg BID vs adalimumab through month 12. Similarly, improvements in MOS-SS domains were generally similar or numerically higher for tofacitinib 5 mg BID vs adalimumab, with some significance observed for tofacitinib 5 mg BID vs adalimumab at some time points; tofacitinib 10 mg BID demonstrated statistical significance vs adalimumab in several domains and at several time points. While together these findings indicate greater sleep improvements with tofacitinib vs adalimumab, the operative mechanism for this treatment difference remains unclear.

Generally, through month 12, improvements in SF-36 PCS and MCS scores, and SF-36 domains were similar or numerically higher with tofacitinib 5 mg BID vs adalimumab, with significance favoring tofacitinib 5 mg BID vs adalimumab observed in SF-36 social functioning domain score at month 3. In contrast, tofacitinib 10 mg BID demonstrated statistical significance vs adalimumab in SF-36 PCS and MCS scores, and several SF-36 domains at several time points. Furthermore, the proportions of patients who reported improvements in SF-36 physical functioning and role-physical domains ≥ MCID, as well as FACIT-F total score ≥ MCID, were also significantly higher with tofacitinib 10 mg BID vs adalimumab at several time points.

At months 3 and 6, significantly higher proportions of patients treated with tofacitinib 10 mg BID reported expected levels (i.e., met or exceeded normative values) for the SF-36 domains of social functioning and mental health vs adalimumab, with significance also seen for the SF-36 domains of role-physical and role-emotional at month 3. Notably, compared with adalimumab, significantly higher proportions of patients reported expected levels of energy (as measured by SF-36 vitality) at months 3 and 6 with tofacitinib 10 mg BID, and at month 12 with both tofacitinib doses.

At month 6, fatigue was moderately to highly correlated with SF-36 domains across active treatment groups, with correlations highest for SF-36 vitality and social functioning. While the correlation between fatigue and MOS-SS Sleep Problems Index I was high across active treatments, correlations between individual MOS-SS domains were generally less pronounced (with somnolence, sleep adequacy, and sleep disturbance the most notable) than those between fatigue and SF-36 domains. Across active treatment groups, disease activity at month 6 was moderately correlated with fatigue and low to moderately correlated with HRQoL measures, including SF-36 general health domain, PCS, and MCS scores. A previous analysis of data from Phase 3 studies of tofacitinib investigated the associations between fatigue and patient-reported disease activity (per Patient Global Assessment of Disease [PtGA]) in patients with RA [36]. In patients receiving tofacitinib 5 or 10 mg BID, correlation analyses demonstrated that improvements from baseline in PtGA were, generally, weakly correlated with improvements from baseline in fatigue at month 3 [36]. Our findings, coupled with this previous analysis, suggest at least a weak to moderate relationship between changes in disease activity and in changes in fatigue. Overall, the correlations observed between measures of fatigue, HRQoL, sleep, and disease activity suggest that patient-reported improvements in symptoms of fatigue and HRQoL may be associated with broader aspects of physical, emotional, and social health in patients with moderate to severe RA, further emphasizing the importance of monitoring fatigue in clinical practice.

Fatigue is increasingly recognized as a debilitating and important symptom of RA and is a major determinant of HRQoL [6, 7]. Currently available treatments for RA improve fatigue to varying degrees, and there is little consensus on treatment for fatigue management in RA [11]. bDMARDs have a moderately beneficial effect on fatigue with no discernible differences between TNFi and non-TNFi bDMARDs [37]. The effectiveness of the bDMARDs suggests an association between fatigue and inflammation [6], and emerging evidence also suggests that Janus kinase (JAK) inhibitors may be more effective than TNFi in this respect [11]. The differences observed between tofacitinib and adalimumab in our post hoc exploratory analysis support this finding, and one possible explanation may be a preferential effect of tofacitinib on multiple inflammatory cytokines through JAK1 and JAK3 inhibition [38, 39], given the association between higher levels of certain cytokines and feelings of tiredness or exhaustion [11]. Evidence from observational cohort studies of patients with early RA suggests that early improvements in disease activity are associated with improvements in fatigue that persist over 5 years of follow-up [13], underscoring the relationship between disease control and HRQoL. We also acknowledge the potential contribution of mood and stress management strategies [15], as well as exercise [40], to the treatment of fatigue, particularly in conjunction with appropriate DMARD therapy for RA.

Noted limitations of this study include the post hoc nature of the analyses. Furthermore, the comparatively small size of the adalimumab treatment group means that results for these patients should be interpreted with caution, as there would have been less statistical power to demonstrate differences vs placebo. Finally, in this analysis, the 13-item FACIT-F was used to evaluate fatigue; however, qualitative data have provided strong support for the validity of a 10-item FACIT-F [3], and it may, therefore, be prudent to employ the 10-item version in future research.

## Conclusions

In this exploratory post hoc analysis of data from over 2000 patients with active RA treated as part of the ORAL clinical trial program, tofacitinib was associated with significantly greater improvements in fatigue, sleep, and HRQoL, including vitality, compared with placebo. Furthermore, in this analysis, some improvements were observed to be significantly greater with tofacitinib vs adalimumab. Correlations between fatigue, PROs of sleep, HRQoL, and disease activity, observed at month 6, underscored the congruence of fatigue and HRQoL with multiple aspects of physical, emotional, and social health.

## Supplementary Information

**Additional file 1: Supplementary Table 1; Supplementary figures 1–2**. Mean change from baseline in PRO domain scores at month 3 across treatment groups; changes from baseline in MOS-SS domains scores up to month 12; changes from baseline in SF-36 domain scores up to month 12.

## Data Availability

Upon request, and subject to review, Pfizer will provide the data that support the findings of this study. Subject to certain criteria, conditions, and exceptions, Pfizer may also provide access to the related individual de-identified participant data. See https://www.pfizer.com/science/clinical-trials/trial-data-and-results for more information.

## Abbreviations

bDMARD: Biologic disease-modifying antirheumatic drug
BID: Twice daily
CATCH: Canadian Early Arthritis Cohort
CDAI: Clinical Disease Activity index
CRP: C-reactive protein
csDMARD: Conventional synthetic disease-modifying antirheumatic drug
DAS28-4(ESR): Disease Activity Score in 28 joints, erythrocyte sedimentation rate
DMARD: Disease-modifying antirheumatic drug
FACIT-F: Functional Assessment of Chronic Illness Therapy-Fatigue
HAQ-DI: Health Assessment Questionnaire-Disability Index
HRQoL: Health-related quality of life
JAK: Janus kinase
LS: Least squares
MCID: Minimum clinically important difference
MCS: Mental Component Summary
MOS-SS: Medical Outcomes Study Sleep scale
PCS: Physical Component Summary
PRO: Patient-reported outcome
PtGA: Patient Global Assessment of Arthritis
Q2W: Once every 2 weeks
RA: Rheumatoid arthritis
SD: Standard deviation
SE: Standard error
SF-36: Short Form-36 Health Survey
TNFi: Tumor necrosis factor inhibitor
VAS: Visual analog scale
