# Supplementary material for: The impact of tofacitinib on fatigue, sleep, and health-related quality of life in patients with rheumatoid arthritis: a post hoc analysis of data from Phase 3 trials
Source: Arthritis Res Ther. 2022 Apr 5;24:83. doi: 10.1186/s13075-022-02724-x (PMC8981846; doi:10.1186/s13075-022-02724-x)
Supplement: Supplementary file 1 — Additional file 1: Supplementary Table 1; Supplementary figures 1–2. Mean change from baseline in PRO domain scores at month 3 across treatment groups; changes from baseline in MOS-SS domains scores up to month 12; changes from baseline in SF-36 domain scores up to month 12. [file 13075_2022_2724_MOESM1_ESM.docx]

**Additional file 1**

**Supplementary Table 1.** Mean change from baseline in PRO domain scores at month 3 across treatment groups^a^

| **Outcome** | **Tofacitinib 5 mg BID**  (*N =*826) | | | **Tofacitinib 10 mg BID**  (*N =*821) | | | **Adalimumab 40 mg Q2W**  (*N =*199) | | | **Placebo**  (*N =*419) | | |
| --- | --- | --- | --- | --- | --- | --- | --- | --- | --- | --- | --- | --- |
|  | n | Mean change | SD | n | Mean change | SD | n | Mean change | SD | n | Mean change | SD |
| **MOS-SS domain scores** | | | | | | | | | | | | |
| Sleep adequacy | 768 | 7.0^***†^ | 27.1 | 773 | 6.8^***^ | 29.1 | 187 | 4.4 | 22.3 | 387 | 0.8 | 25.7 |
| Awaken short of breath/with headache | 768 | –0.6 | 24.5 | 773 | –1.8^*^ | 22.0 | 187 | –2.5 | 21.6 | 387 | 0.8 | 21.4 |
| Sleep disturbance | 767 | –7.5^*^ | 19.5 | 772 | –8.2^**†^ | 21.5 | 187 | –6.6 | 20.3 | 387 | –4.0 | 20.8 |
| Sleep quantity (hours) | 771 | 0.2^*^ | 1.5 | 771 | 0.3^***^ | 1.3 | 186 | 0.3 | 1.4 | 386 | 0.0 | 1.4 |
| Snoring | 761 | –1.7 | 23.4 | 771 | 0.0 | 21.3 | 186 | –0.3 | 20.2 | 385 | –0.7 | 23.2 |
| Somnolence | 767 | –5.6^***^ | 19.6 | 773 | –6.0^***†^ | 20.5 | 186 | –2.1 | 18.3 | 387 | –0.2 | 19.7 |
| **SF-36 domain scores** | | | | | | | | | | | | |
| Physical functioning | 772 | 4.7^***^ | 8.7 | 774 | 6.8^***††^ | 8.9 | 188 | 4.9^**^ | 9.0 | 389 | 1.9 | 8.6 |
| Role-physical | 773 | 5.4^***^ | 9.5 | 775 | 7.4^***††^ | 10.0 | 188 | 4.8^***^ | 8.7 | 389 | 2.0 | 8.8 |
| Bodily pain | 772 | 7.2^***^ | 8.9 | 775 | 9.1^***††^ | 9.6 | 188 | 7.7^***^ | 9.4 | 389 | 2.7 | 8.5 |
| General health | 772 | 4.6^***^ | 8.2 | 774 | 5.3^***^ | 7.7 | 187 | 4.5^***^ | 7.4 | 389 | 1.0 | 7.0 |
| Vitality | 773 | 5.9^***^ | 9.5 | 775 | 6.4^***†^ | 9.8 | 188 | 5.7^***^ | 8.6 | 389 | 1.5 | 8.5 |
| Social functioning | 773 | 5.3^***†^ | 10.5 | 775 | 6.1^***†††^ | 11.2 | 188 | 4.3 | 9.1 | 389 | 1.5 | 10.4 |
| Role-emotional | 772 | 3.8^**^ | 12.2 | 774 | 6.3^***†^ | 13.2 | 188 | 3.7^*^ | 12.2 | 388 | 1.3 | 11.8 |
| Mental health | 773 | 4.1^***^ | 10.3 | 775 | 5.0^***^ | 10.3 | 188 | 3.7^*^ | 9.5 | 389 | 0.9 | 9.5 |

Data were pooled from Phase 3 ORAL Scan, ORAL Standard, and ORAL Sync study datasets, and are presented for the full analysis set

^a^All treatments were administered in combination with background conventional synthetic disease-modifying antirheumatic drugs
The *p*-values were derived from LS means, which were based on a model controlling for baseline values

^*^*p* < 0.05, ^**^*p* < 0.01, and ^***^*p* < 0.001 for tofacitinib and adalimumab vs placebo; ^†^*p* < 0.05, ^††^*p* < 0.01, and ^†††^*p* < 0.001 for tofacitinib vs adalimumab

BID, twice daily; LS, least squares; MOS-SS, Medical Outcomes Study Sleep scale; PRO, patient-reported outcome; Q2W, once every 2 weeks; SD, standard deviation; SF-36, Short Form-36 Health Survey

**Supplementary Fig. 1.** Changes from baseline in MOS-SS domains scores up to month 12

LS mean change from baseline in MOS-SS domain scores to month 12 across treatment groups^a^ pooled from Phase 3 ORAL Scan, ORAL Standard, and ORAL Sync study datasets (full analysis set)


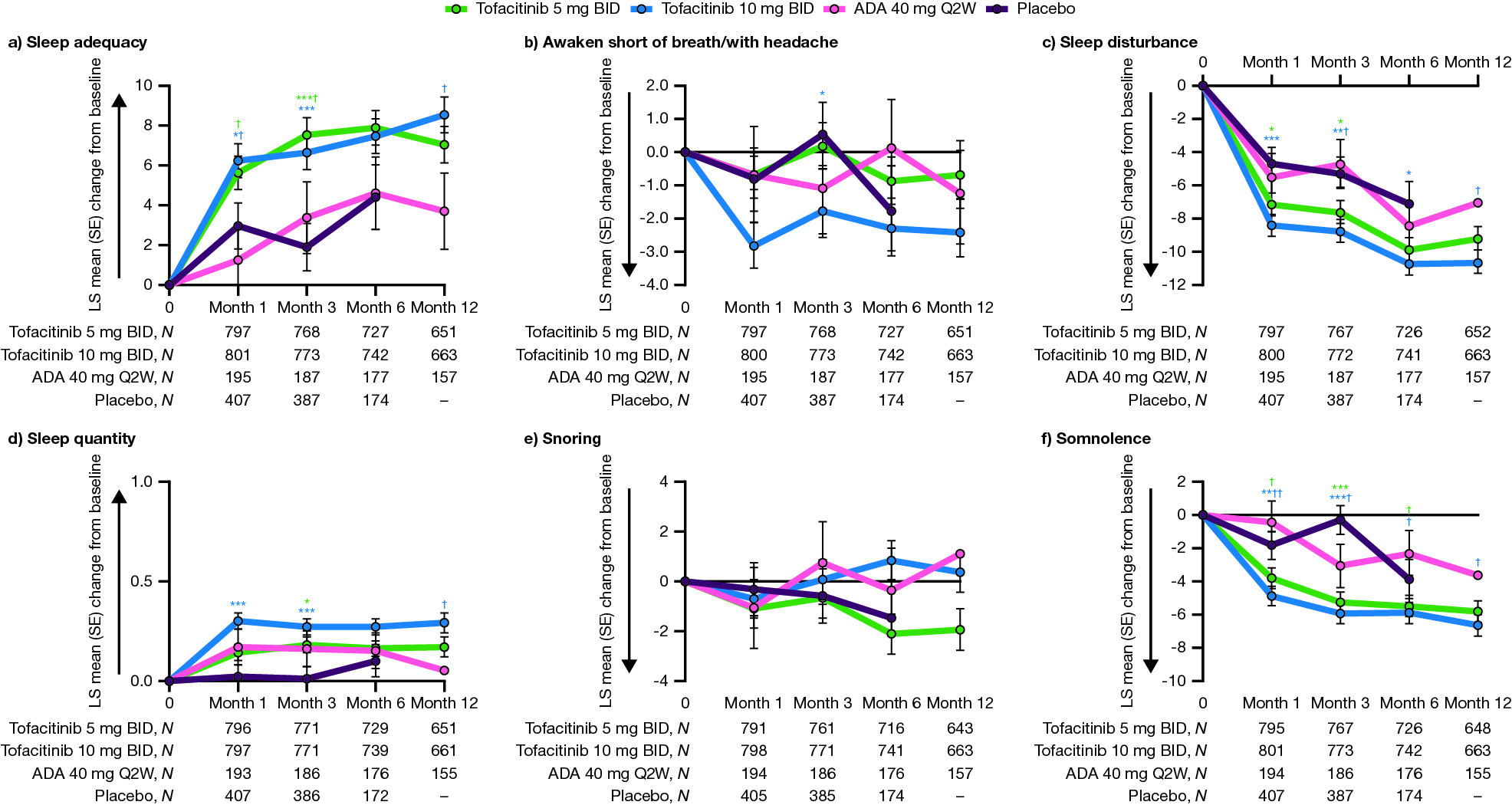


^a^All treatments were administered in combination with background conventional synthetic disease-modifying antirheumatic drugs

^*^*p* < 0.05, ^**^*p* < 0.01, and ^***^*p* < 0.001 for tofacitinib and adalimumab vs placebo; ^†^*p* < 0.05, ^††^*p* < 0.01, and ^†††^*p* < 0.001 for tofacitinib vs adalimumab

The arrows on the y-axes indicate the direction of improvement

ADA, adalimumab; BID, twice daily; LS, least squares; MOS‑SS, Medical Outcomes Study Sleep scale; Q2W, once every 2 weeks; SE, standard error

**Supplementary Fig. 2.** Changes from baseline in SF-36 domain scores up to month 12

LS mean change from baseline in SF-36 domain scores to month 12 across treatment groups^a^ pooled from Phase 3 ORAL Scan, ORAL Standard, and ORAL Sync study datasets
(full analysis set)

**^
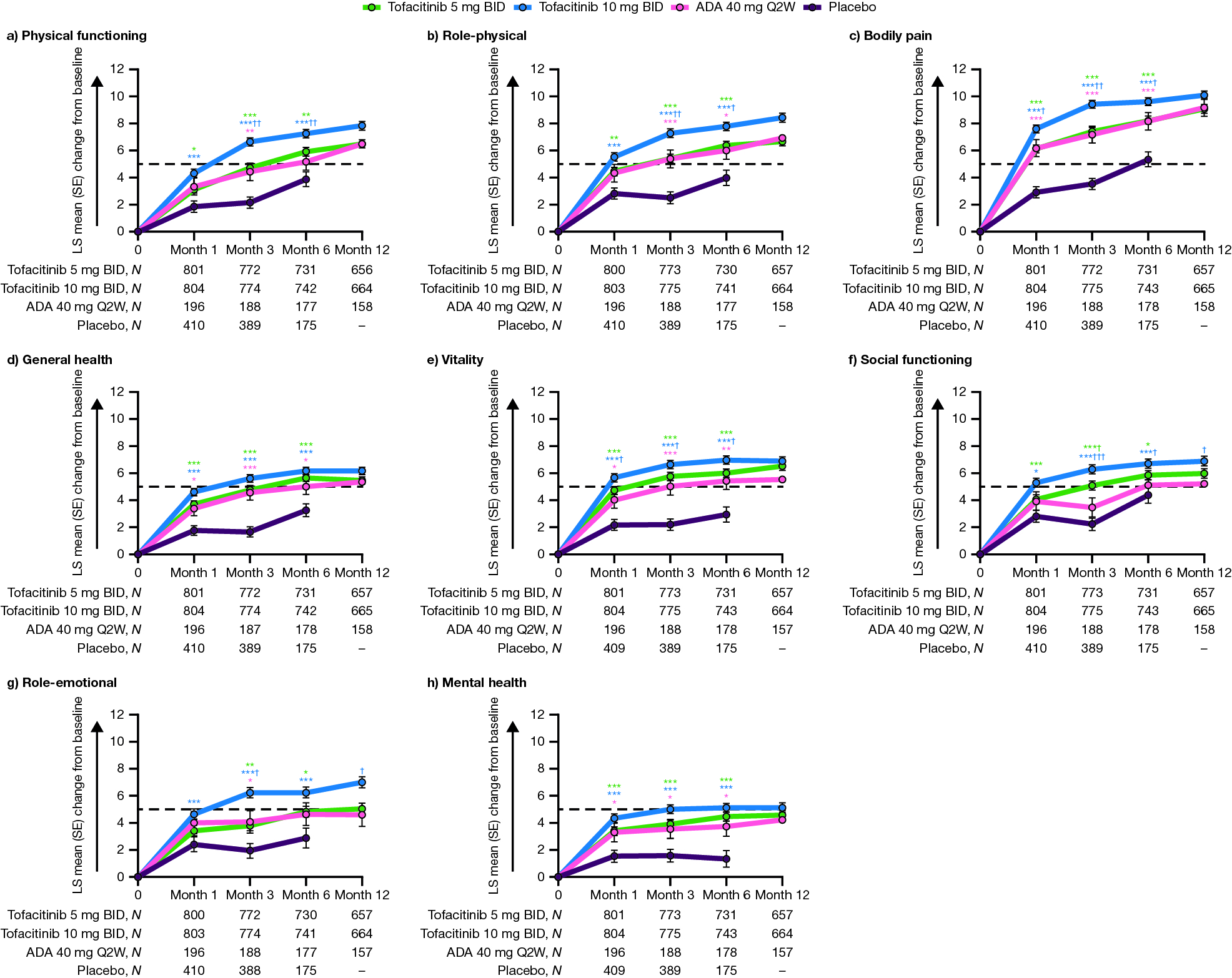
^**

^a^All treatments were administered in combination with background conventional synthetic disease-modifying antirheumatic drugs

^*^*p* < 0.05, ^**^*p* < 0.01, and ^***^*p* < 0.001 for tofacitinib and adalimumab vs placebo; ^†^*p* < 0.05, ^††^*p* < 0.01, and ^†††^*p* < 0.001 for tofacitinib vs adalimumab

The horizontal dashed lines represent the MCIDs

The arrows on the y-axes indicate the direction of improvement

ADA, adalimumab; BID, twice daily; LS, least squares; MCID, minimum clinically important difference; Q2W, once every 2 weeks; SE, standard error; SF-36, Short Form-36 Health Survey
